# Free radical sensors based on inner-cutting graphene field-effect transistors

**DOI:** 10.1038/s41467-019-09573-4

**Published:** 2019-04-04

**Authors:** Zhen Wang, Kongyang Yi, Qiuyuan Lin, Lei Yang, Xiaosong Chen, Hui Chen, Yunqi Liu, Dacheng Wei

**Affiliations:** 1 State Key Laboratory of Molecular Engineering of Polymers, Fudan University, 200433 Shanghai, China; 2 Department of Macromolecular Science, Fudan University, 200433 Shanghai, China; 3 Institute of Molecular Materials and Devices, Fudan University, 200433 Shanghai, China; 4 Department of Chemistry, Fudan University, 200433 Shanghai, China

## Abstract

Due to ultra-high reactivity, direct determination of free radicals, especially hydroxyl radical (•OH) with ultra-short lifetime, by field-effect transistor (FET) sensors remains a challenge, which hampers evaluating the role that free radical plays in physiological and pathological processes. Here, we develop a •OH FET sensor with a graphene channel functionalized by metal ion indicators. At the electrolyte/graphene interface, highly reactive •OH cuts the cysteamine to release the metal ions, resulting in surface charge de-doping and a current response. By this inner-cutting strategy, the •OH is selectively detected with a concentration down to 10^−9^ M. Quantitative metal ion doping enables modulation of the device sensitivity and a quasi-quantitative detection of •OH generated in aqueous solution or from living cells. Owing to its high sensitivity, selectivity, real-time label-free response, capability for quasi-quantitative detection and user-friendly portable feature, it is valuable in biological research, human health, environmental monitoring, etc.

## Abstract (web summary)

Field effect transistors based on graphene hold promise for future sensing applications. Here, the authors report a free radical sensor in which the •OH radical cuts cysteamine to release Cd^2^^+^ from the electrolyte/graphene interface and selectively detects •OH with a concentration down to 10^−9^ M.

## Introduction

Detection of the free radical is of great importance for human health, as the free radical is generally believed to play important roles in the pathogenesis of various human diseases^1^. For instance, overproduced reactive oxygen species (ROS) cause oxidative stress through the oxidation of biomolecules, such as lipids, proteins, and DNA, in cells and tissues^1,2^. Among them, hydroxyl radical (•OH), as one of the most reactive chemical species known, induces large aggression to human health due to its ultra-high reactivity with various biological species compared with other ROS^3–5^. The •OH can damage the bases of DNA and mediate redox alteration of cell-membrane Ca^2+^ channels. Therefore, the fast and real-time monitoring strategies for physiologically important free radicals, especially the •OH, is of great significance. Till now, analytical methods are still the bottleneck for progress in understanding physiological and pathological events such as aging, cancer, ischemia/reperfusion injury, traumatic brain injury, etc.^1,6,7^. In order to understand the role that free radical plays in biological and pathological events, much attention has been paid on monitoring the free radicals in living systems. Traditionally, the free radical is detected by electron spin resonance (ESR) spectroscopy^8^, fluorescence spectroscopy^9–11^, chromatography^12,13^, and electrochemical approaches^14^. However, these techniques suffer from some of the disadvantages such as costly instrumentation, low throughput, complicated sample preparation, need for well-trained operators, or lack of portability. Moreover, these techniques normally require spin traps or fluorescent probes, which introduce additional contaminations that are involved in the detection system and interfere with the desired signals^8–14^. The spin traps or fluorescent probes determine the detection limit and selectivity. In some cases, the detection limit of •OH is only 10^−6^ M (fluorescence)^9,11^, while ROS such as •O_2_^−^ and •HO_2_ can also induce a response in the fluorescence or ESR signal^10,15^. Therefore, it is still a challenge to real-time monitor the free radicals with high sensitivity and selectivity by a low-cost, portable, and user-friendly analytical platform.

As a promising detection technique, field-effect transistor (FET)-based sensor works by transducing and monitoring the absorbate-induced perturbations into the conductance change in the channel materials, typically in terms of the source-drain current^16,17^. The channel material with high surface-to-volume ratio is favorable generally since it implies higher adsorption site density available^18^. Owing to its high carrier mobility and atomic thickness, graphene bears ultra-high sensitivity to electrical perturbations from the external environment^17,19–22^, which has been integrated into FET sensors with advantages such as label-free detection, high stability, flexibility, fast response, biological compatibility, and user-friendship^23^, compared with other detection techniques. In a graphene-based FET sensor, the graphene is normally surface functionalized, and the specific interaction between the functional group and the analyte allows the selective detection of the pH^24^, DNA^25^, RNA^26^, living cells^27,28^, gas^29^, metal ions^30–32^, etc. Although the free radical has a strong doping effect to graphene^33,34^, it is still challenging to directly monitor the free radical in the aqueous environment by the FET sensors. One pioneering work^35^ develops a poly(3-hexylthiophene) FET sensor functionalized with rutin. The oxidation of rutin by superoxide (•O_2_^−^) induces current perturbations and enables the detection of •O_2_^−^. However, compared with •O_2_^−^ or other ROS, the highly reactive •OH has ultra-short lifetime. The lifetime of •OH is around 3 × 10^−6^ s^36^, 6 orders of magnitude shorter than that of •O_2_^−^ (~1 s)^37^. Considering the ultra-short lifetime and the relatively low rate constant of the oxidation reaction (i.e., 1.5 × 10^6^ M^−1^ s^−1^ for the reaction between •O_2_^−^ and rutin)^38^, the •OH is easy to convert to H_2_O_2_ or other ROS without reaction with the functional groups. Till now, a FET sensor that can monitor highly reactive free radicals like •OH is still absent. Moreover, besides the pH value, quantitative analysis of the chemicals is difficult for a FET sensor, hampering its practical applications, especially in the bio-system.

Herein we develop a FET sensor that can selectively detect highly reactive free radicals. The FET sensor has a graphene channel functionalized with metal ion indicators via cysteamine as the inner connector. Owing to the ultra-fast reaction between •OH and thiol derivatives (rate constant: ~10^10^ M^−1^ s^−1^)[5], the cysteamine can be selectively cut by the •OH despite of its ultra-short lifetime^39–41^. At the electrolyte/graphene interface, metal ions are released from graphene, leading to a surface charge de-doping and a current response in the channel. Thus, by monitoring the current perturbations induced by the metal ions, the inner-cutting strategy enables indirect detection of •OH with a concentration down to 10^−9^ M. The label-free real-time detection has high selectivity to •OH over metal ions or other ROS interferents. The sensitivity can be easily tuned by modifying the graphene channel with different amounts of metal ions, and then quasi-quantitative detection of •OH is realized when measuring the sample by using FET sensors with different sensitivities. As an application, the FET sensors were used in quasi-quantitative detection of •OH generated from Hela cells, showing great potential of the inner-cutting strategy for practical applications in monitoring free radicals or other analytes with ultra-high reactivity and ultra-short lifetime.

## Results

### Device fabrication and the •OH detection

**Fig. 1:**
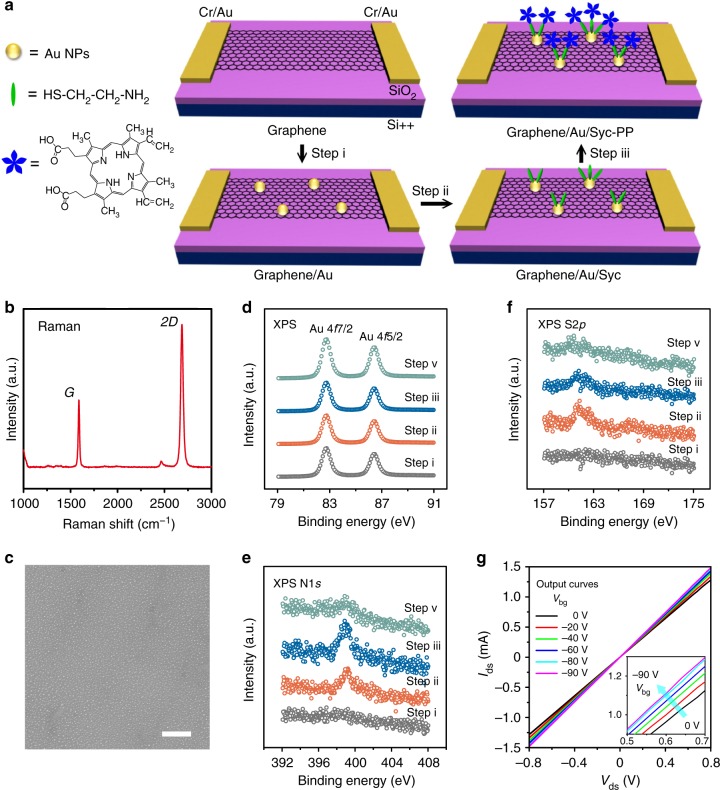
Fabrication and characterization of a field-effect transistor (FET) sensor. **a** Schematic diagram of the device fabrication process. **b** Raman spectrum of a graphene film. **c** Field-emission scanning electron microscope images of the graphene film decorated with Au nanoparticles (step i). **d**–**f** X-ray photoelectron spectroscopic spectra of Au 4*f*_7/2_ and Au 4*f*_5/2_, S 2*p*, and N 1*s* for (step i) graphene/Au, (step ii) graphene/Au/Cys, (step iii) graphene/Au/Cys-PP, and (step v) graphene/Au/Cys-PP-Cd^2+^ after reaction with •OH. **g** Output characteristics of a graphene/Au/Cys-PP FET sensor (*V*_bg_ varies from 0 to −90 V). The scale bar in **c** is 200 nm

The fabrication procedure of the FET sensor is schematically shown in Fig. 1a. Briefly, a monolayer graphene produced by chemical vapor deposition (CVD; Supplementary Note 1) is transferred onto a SiO_2_/Si substrate, where graphene acts as the sensing channel, SiO_2_ as the gate dielectric, and Si as the back gate. Raman spectrum (Fig. 1b) shows a tiny *D* peak, a strong and sharp 2*D* peak compared with the *G* peak, indicating the high quality and the monolayer nature of the graphene. And then Cr/Au (5/50 nm thickness) is patterned on graphene by photolithography as the electrodes. Au nanoparticles (NPs) are deposited onto the graphene channel by vacuum evaporation and thermal annealing (step i). The resulting channel is denoted as graphene/Au. Field-emission scanning electron microscopic (FESEM) and transmission electron microscopic (TEM) images reveal that the Au NPs are uniformly distributed on the graphene surface without agglomeration (Fig. 1c, Supplementary Fig. 1). Atomic force microscopic (AFM) images show that the height of the Au NPs is around 4 nm (Supplementary Fig. 2a). The van-der-Waals binding between Au NPs and graphene is strong enough to retain Au NPs in place even after several cycles of washing and drying. After that, the device is submerged in 10^−3^ M cysteamine solution in order to assemble a monolayer of cysteamine on Au NPs (graphene/Au/Cys) through Au−S bonds (step ii). Finally, protoporphyrin IX (PP) is immobilized on the cysteamine (graphene/Au/Cys-PP) (step iii). The corresponding AFM image shows that the height of the Au NPs increases to around 6 nm (Supplementary Fig. 2b), indicating the successful modification of Cys-PP onto Au NPs. To characterize the chemical structure, the sample is measured by X-ray photoelectron spectroscopy (XPS, Fig. 1d–f) after each step. Au 4*f*_7/2_ and Au 4*f*_5/2_ peaks located at about 82.7 and 86.4 eV appear after step i, which are ascribed to Au NPs on the graphene. S 2*p* (164.2 eV) and N 1*s* (400.4 eV) peaks appear after step ii, indicating immobilization of cysteamine onto graphene/Au. A slight increase of N 1*s* peak and decrease of S 2*p* are observed after step iii. The result implies the successful assembly of PP, since PP contains high proportion of N without S. In contrast, Cys-PP cannot be directly linked to graphene surface as no N 1*s* or S 2*p* peaks appear on bare graphene after step iii (Supplementary Fig. 3). Moreover, an obvious peak at 403 nm appears in ultraviolet-visible (UV-vis) absorption spectrum after step iii, confirming that the PP is successfully modified on the channel (Supplementary Fig. 4).

**Fig. 2:**
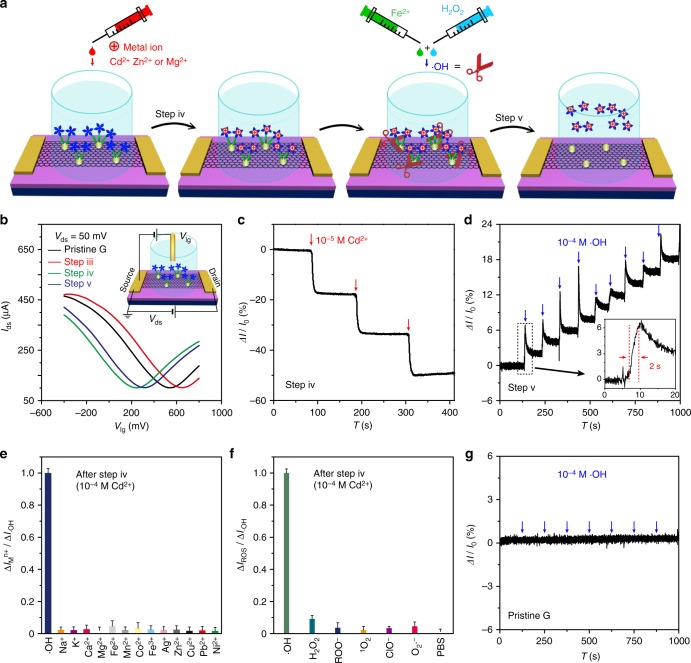
Detection performance and mechanism of the field-effect transistor (FET) sensor. **a** Schematic diagram of the •OH detection. **b** Liquid gate transfer curves of a FET device of pristine graphene (black), after step iii (red), after step iv (10^−4^ M Cd^2+^, green), and after step v (10^−4^ M •OH, blue), when liquid gate voltage (*V*_lg_) varies from −400 to 800 mV. **c**, **d** Real-time electrical responses of a graphene/Au/Cys-PP FET device upon successive addition of 1 × 10^−5^ M Cd^2+^ (step iv) and subsequent 1 × 10^−4^ M •OH (step v). **e** Selectivity of a graphene/Au/Cys-PP-Cd^2+^ FET sensor (modified in 10^−4^ M Cd^2+^) toward •OH (10^−6^ M) and metal ions (Na^+^, K^+^, Ca^2+^, Mg^2+^, Fe^2+^, Mn^2+^, Co^2+^, Fe^3+^, Ag^+^, Zn^2+^, Cu^2+^, Pb^2+^, Ni^2+^, 10^−6^ M). **f** Selectivity of a graphene/Au/Cys-PP-Cd^2+^ FET sensor (modified in 10^−4^ M Cd^2+^) toward •OH (10^−6^ M) and other reactive oxygen species (H_2_O_2_, ROO^−^, ^1^O_2_, ClO^−^, O_2_•^−^, phosphate-buffered saline, 10^−6^ M). **g** The response of a pristine graphene channel (without modification) upon successive addition of 1 × 10^−4^ M •OH. The error bars are defined by the standard deviation of the results from three parallel experiments

To obtain a solution-based FET sensor (Figs. 1g and 2a, b), a chamber is fabricated on the channel, which is employed for all solution-based measurements. The electrodes are located beside the chamber with channel length around 8 mm. After dipping in 1 × 10^−5^ M Cd^2+^ aqueous solution, Cd^2+^ ions are bound to the channel surface to charge-dope graphene (Fig. 2c) (graphene/Au/Cys-PP-Cd^2+^)^42,43^. In the process, the drain-source current (*I*_ds_) rapidly decreases upon successive addition of Cd^2+^. After coordination with Cd^2+^ (step iv), the *I*_ds_ of the graphene/Au/Cys-PP-Cd^2+^ channel (Fig. 2d) shows a rapid response upon exposure to 1 × 10^−4^ M •OH within 2 s (step v), thus the graphene/Au/Cys-PP-Cd^2+^ FET works as a real-time label-free •OH sensor. The current changes (Supplementary Fig. 5) are detectable even when the concentration of •OH decreases to 10^−7^ M.

The complexity of biological systems presents a great challenge in selective detection of •OH. The selectivity of the graphene/Au/Cys-PP-Cd^2+^ FET sensor is evaluated by monitoring the current signals induced by metal ions (Fig. 2e) and other potential interferents (Fig. 2f) that may coexist in biological systems. The low response to metal ions is an indication of high quality of the graphene^44–46^, as no binding with ions is expected and the pristine graphene is intrinsically insensitive to outside metal ions or pH variance owing to the ideal surface with pure saturated carbon bonds. When exposed to possible ROS interferents, such as H_2_O_2_, a little current change is observed. The negligible response (<10%) upon the same concentration of metal ions or ROS over •OH suggests the high selectivity of the present sensor for detecting •OH against metal ions and other coexisting biological molecules. Moreover, the storage stability of the graphene/Au/Cys-PP-Cd^2+^ FET sensor is investigated by monitoring the current response to 10^−4^ M •OH three times daily after storage in a refrigerator, and the current response when adding 10^−4^ M •OH still retains >94.5% of its initial response after 1 week of storage (Supplementary Fig. 6).

**Fig. 3:**
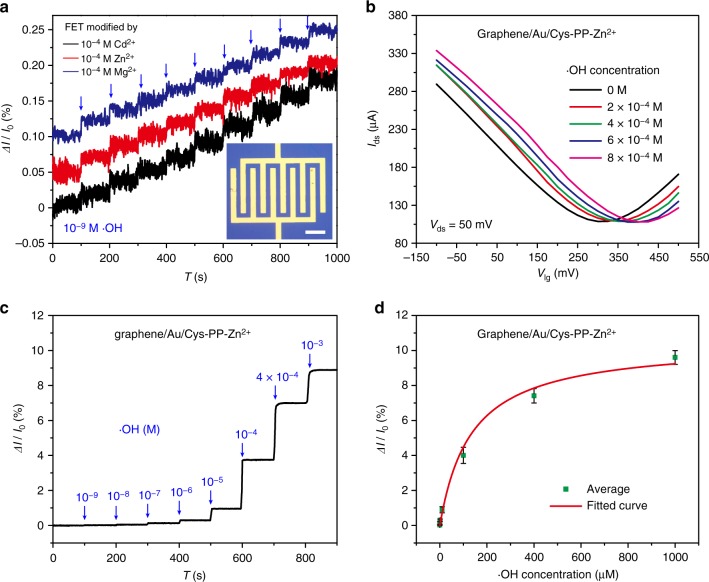
The field-effect transistor (FET) sensor with Cd^2+^, Zn^2+^, and Mg^2+^ as the indicator. **a** Real-time electrical responses of a graphene/Au/Cys-PP-metal ion FET device (modified in 10^−4^ M Cd^2+^, Zn^2+^, or Mg^2+^) upon successive addition of 10^−9^ M •OH. The inset shows the FET device with interdigital electrodes. **b** Liquid gate transfer curves (*V*_ds_ = 50 mV) of a graphene/Au/Cys-PP-Zn^2+^ FET device (modified in 10^−4^ M Zn^2+^) before and after addition of 2 × 10^−4^, 4 × 10^−4^, 6 × 10^−4^, and 8 × 10^−4^ M •OH, when *V*_lg_ varies from −100 to 500 mV. **c** Real-time response upon various concentrations of •OH (from 10^−9^ to 10^−4^ M) for the graphene/Au/Cys-PP-Zn^2+^ FET device (modified in 10^−4^ M Zn^2+^). **d** The electrical responses versus the •OH concentration for the graphene/Au/Cys-PP-Zn^2+^ FET device (modified in 10^−4^ M Zn^2+^). The scale bar in **a** is 200 μm. The error bars are defined by the standard deviation of the results from three parallel experiments

Besides Cd^2+^, other metal ions such as Zn^2+^ and Mg^2+^, which can coordinate with PP, are also used as indicators to modify the graphene/Au/Cys-PP FET device, thus the biological compatibility can be improved as a result of avoiding the usage of heavy metal ions. Considering the small leakage current with a fluctuation around 1 nA (<0.0005% of the *I*_ds_) in 0.01× phosphate-buffered saline (PBS; Supplementary Fig. 7), the interdigital electrodes (50 μm channel length, Fig. 3a) can directly be used as the source-drain electrodes in the FET sensor. Owing to the small channel length, an improved sensitivity is achieved and the current response can still be detectable after modifying the graphene/Au/Cys-PP FET device with 10^−4^ M Cd^2+^, 10^−4^ M Zn^2+^, or 10^−4^ M Mg^2+^, when the concentration of •OH decreases to 10^−9^ M (Fig. 3a, Supplementary Figs. 8 and 9).

### Free radical detection mechanism

FET is a device in which a gate electric field controls the current flow through the conducting channel^47^. When operating in solution, the gate electric field can be applied via the electrolyte/semiconductor interface, where an electrical double-layer exists that enables fast device response and low voltage operation^20^. Figure 1g shows the output curves of a graphene/Au/Cys-PP FET device at different back-gate voltages (*V*_bg_). The linear nature of the *I*_ds_ versus drain-source voltage (*V*_ds_) curve reveals an ideal Ohmic contact between the channel and the electrode, and such reliable contact provides the possibility of the application in sensitive sensors. The Ohmic contact remains at different *V*_bg_, suggesting that the current changes are mainly affected by the electrostatic gate doping effect, rather than the contact resistance. The *I*_ds_ versus liquid gate voltage (*V*_lg_) (liquid gate transfer curve, *V*_ds_ = 50 mV, Fig. 2b) shows that the FET device has an ambipolar property. The Dirac point of the pristine graphene is located at around 540 mV and the *I*_ds_ increases by about 360 μA when negatively increasing *V*_lg_ from 540 to −400 mV. Owing to the chemical doping effect, the Dirac point shifts to around 650 mV after modification with Au NPs and Cys-PP. Near zero gate voltage, the graphene/Au/Cys-PP channel shows a typical p-type behavior. Therefore, real-time responses in this study are measured in the p-type region.

The FET sensor is the combination of a sensor and an amplifier, in which a small potential alteration may induce a pronounced change of channel current^16,48^. The sensing process takes place at the electrolyte/semiconductor interface. The binding of charged species or the charge transfer at the interface lead to a variation of charge or electric potential of the conducting channel, in a way similar to applying an external potential to the gate electrode in a conventional FET device, leading to electrical conductivity changes associated with the analyte in real time^16,48^. Graphene combines extremely high charge mobility with atomic thickness, all the charge carriers in graphene flow solely on the surface and expose directly to the sensing environment, and thus the electrical property of graphene after appropriate modification is highly sensitive to the minute specific electrochemical perturbations imposed by the surrounding environment in solution. However, some free radicals like •OH have high reactivity and ultra-short lifetime. The lifetime of •OH is only around 3 × 10^−6^ s^36^, orders of magnitude shorter than normal free radicals like •O_2_^−^ (~1 s in aqueous solution)^37^, •NO (0.09 to >2 s)^49^, etc. The •OH is easy to convert to H_2_O_2_ or other ROS without arising current responses in the channel. Thus it is still difficult to directly monitor the highly reactive free radicals like •OH in the aqueous environment, although these radicals have a doping effect to graphene^33,34^. The control experiment also shows that no critical changes in the current are detected (Fig. 2g), when successively adding 1 × 10^−4^ M •OH on a pristine graphene FET device.

To solve this problem, an inner-cutting strategy (Fig. 2a) is developed to achieve an indirect label-free detection of •OH. The graphene channel is functionalized with metal ions via Au-S bonds of the cysteamine. The •OH reacts with thiol derivatives with rate constant in the range of 10^9^–10^10^ M^−1^ S^−1^, >4 orders of magnitude faster than other ROS, such as H_2_O_2_ (0.9 M^−1^ S^−1^)^5^ and •O_2_^−^ (10^4^–10^5^ M^−1^ S^−1^)^50^. After the reaction, the XPS spectra of the graphene/Au/Cys-PP-Cd^2+^ (Fig. 1d–f) show that the Au 4*f* peaks maintain while the S 2*p* and N 1*s* peaks disappear, indicating successful cutting of Cys-PP-Cd^2+^ from Au NPs. Thus the ultra-fast reaction enables selective cut of the cysteamine by •OH and releases metal ions in spite of the ultra-short lifetime of •OH, leading to a controllable surface charge doping or de-doping of the graphene. Figure 2c and Supplementary Figs. 10 and 11 display the real-time responses of the graphene/Au/Cys-PP FET device during a cumulative addition of Cd^2+^ or Zn^2+^. The current decreases with increasing metal ion concentration and reaches saturation until the concentration increase to ~10^−4^ M. The Dirac point of the graphene/Au/Cys-PP FET negatively shifts by 420 mV after modification in 10^−4^ M Cd^2+^ (Fig. 2b), which is attributed to the coordination reaction of Cd^2+^ with the PP on the graphene channel. The corresponding charge density of the bonded Cd^2+^ is estimated to be 0.1 nm^−2^ (Supplementary Note 2). Considering the fact that the distance between Cd^2+^ and graphene is within the Debye length (Supplementary Note 3)^51^, the positive electrical field of the bonded Cd^2+^ forces the positively charged holes away from the electrolyte/graphene interface owing to the electrostatic gating effect. Thus it decreases the hole concentration in the graphene sheet and leads to negative current response of the graphene/Au/Cys-PP FET device^31,52^. When the •OH is added into the electrolyte by Fenton reaction, the self-assembled monolayer of cysteamine is destroyed by the highly reactive •OH, leading to release of metal ions from the electrolyte/graphene interface. Owing to surface charge de-doping, the current recovers (Figs. 2d and 3a, Supplementary Figs. 8 and 9), and the Dirac point of the graphene/Au/Cys-PP-Cd^2+^ FET positively shifts by 57 mV upon addition of 10^−4^ M •OH (Fig. 2b), thus the •OH radicals are indirectly detected. The device exhibits ultrahigh sensitivity to the concentration change of the metal ions. Remarkable response is obtained even when the concentration change of Cd^2+^ decreases to 10^−10^ M (Supplementary Fig. 14). The sensitivity of the graphene/Au/Cys-PP FET to the concentration change of Cd^2+^ is among the highest values of graphene-based FET metal ion sensors^17,19–22,30–32^, which brings the possibility of the indirect detection of •OH with high sensitivity.

To prove the inner-cutting mechanism, we measured the graphene/Au/Cys-PP-Cd^2+^ FET, the graphene/Au/Cys-PP-Zn^2+^ FET, and the graphene/Au/Cys-PP-Mg^2+^ FET upon successive addition of 2 × 10^−4^ M •OH. Both the liquid gate transfer curves and the back gate transfer curve (Fig. 3b, Supplementary Figs. 12 and 13) gradually shift positively and the current reaches saturation until the •OH concentration increases to ~10^−3^ M (Fig. 3c). Percentage change in *I*_ds_ (Δ*I*/*I*_0_) increases stepwise at each concentration. Figure 3d shows Δ*I*/*I*_0_ plotted as a function of the •OH concentration from 10^−9^ to 10^−3^ M, which can be described by the Hill–Langmuir equation^19,53,54^:

1 $$\frac{{{\mathrm{\Delta I}}}}{{I_0}} = \frac{{\left( {\frac{{{\mathrm{\Delta I}}}}{{I_0}}} \right)_{{\mathrm{max}}}C_{{\mathrm{ \bullet OH}}}}}{{K_{\mathrm{D}} + C_{{\mathrm{ \bullet OH}}}}}$$

where (Δ*I*/*I*_0_)_max_ is the saturated value of the Δ*I*/*I*_0_, *C*_•OH_ is the •OH concentration, and *K*_D_ is a constant related to the ability of the •OH to cut the Au-S bonds. The *K*_D_ value is extracted from the fitted curve to be about 9.95 μM, indicating the relatively high reactivity between •OH and Au-S bonds.

The selective detection of •OH is attributed to its high reactivity among the ROS. The redox potential of •OH is 2.31 V, higher than other ROS such as •O_2_^−^ (0.94 V), H_2_O_2_ (0.32 V), and ROO• (0.77 V)^5^. The redox potential is defined compared with H_2_/H^+^ to represent the affinity of a substance for electrons. Higher potential means larger inclination to obtain electrons and stronger oxidizability, thus the cysteamine is inclined to react with •OH rather than other ROS. This, as well as higher reaction rate constant of •OH with the cysteamine compared with other ROS, gives rise to selective reaction of •OH and cysteamine and endows the FET sensor with fast response and high selectivity (Fig. 2e, f).

### Quasi-quantitative detection

**Fig. 4:**
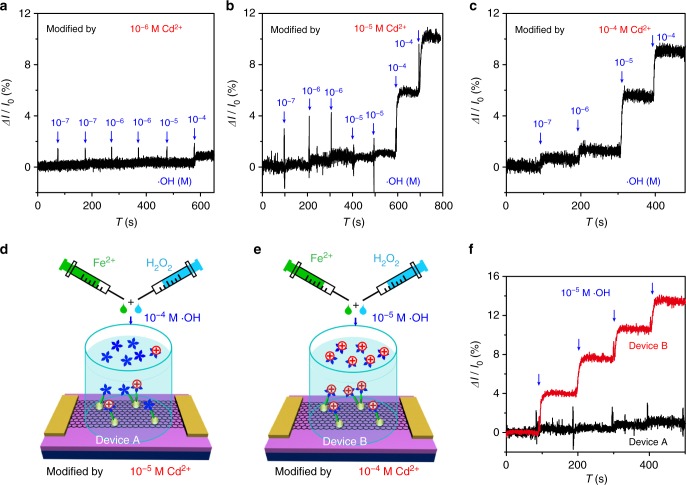
Quasi-quantitative detection of •OH. **a**–**c** Real-time response upon various concentrations of •OH (from 10^−7^ to 10^−4^ M) for the field-effect transistor (FET) sensors with channel doped with **a** 10^−6^ M Cd^2+^, **b** 10^−5^ M Cd^2+^, or **c** 10^−4^ M Cd^2+^. **d**, **e** Schematic diagram of the detection of •OH using the FET sensors modified by different concentrations of Cd^2+^. **f** Real-time response upon successive addition of 10^−5^ M •OH for the FET sensors with channel doped with 10^−5^ M Cd^2+^ (black, Device A) and 10^−4^ M Cd^2+^ (red, Device B)

Non-linear response of the current to different concentrations of •OH (Fig. 3c, d) indicates that the graphene/Au/Cys-PP-metal ion FET, similar to other FET sensors^19–22^, cannot be directly used in quantitatively monitoring the •OH. However, the high controllability of the surface charge doping by metal ions allows a quasi-quantitative detection. Figure 4 shows the real-time current response to different concentrations of •OH, after exposing the graphene/Au/Cys-PP FET devices to aqueous solutions with 10^−4^, 10^−5^, or 10^−6^ M Cd^2+^, respectively. 10^−4^ M •OH is detectable when the device has been exposed to 10^−6^ M Cd^2+^ (Fig. 4a), while the detectable concentration decreases to 10^−7^ M •OH after exposing the device to 10^−4^ M Cd^2+^ (Fig. 4c). The increased •OH sensitivity should be attributed to the enhanced surface doping or de-doping effect. It is because that, after exposure in high concentration metal ions, more positively charged metal ions are bonded on the graphene surface. As a result, when a certain concentration of •OH is added, more metal ions are released from the electrolyte/graphene interface and larger current response is expected. Therefore, the sensitivity of the •OH FET can be tuned by the amount of surface-bonded metal ions.

The quasi-quantitative detection is achieved by monitoring the •OH using FET sensors with different sensitivities. For instance, two graphene/Au/Cys-PP FET devices are exposed in 10^−5^ M (Fig. 4d) and 10^−4^ M Cd^2+^ (Fig. 4e), respectively, resulting in FET sensors with different •OH sensitivities. When adding 10^−5^ M •OH solution onto the devices, the sensors exhibit different real-time responses (Fig. 4f). The current of the device exposed in 10^−4^ M Cd^2+^ varies by ~4%. According to the result in Fig. 4c, the •OH concentration should be ≥10^−5^ M. In the case of the device exposed in 10^−5^ M Cd^2+^, the current response is <1%. The •OH concentration should be ≤10^−5^ M. Therefore, the •OH concentration should be at a level of 10^−5^ M, and a quasi-quantitative detection is realized with the aid of controllable surface charge doping by metal ions.

### Detection of •OH produced by living cells

**Fig. 5:**
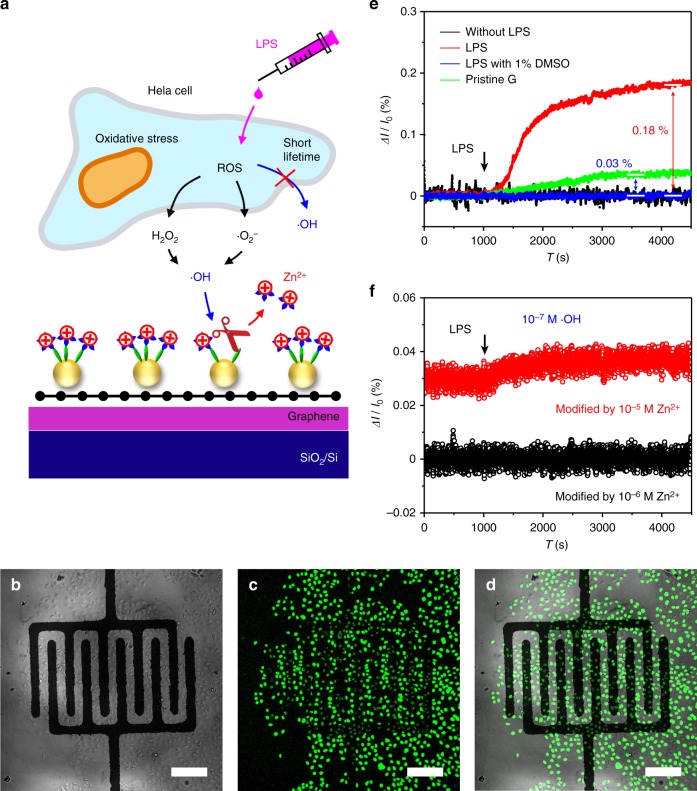
Real-time detection of •OH in living system. **a** Schematic diagram of the detection of •OH produced by Hela cells. **b** Bright field image, **c** confocal fluorescence image (excited by 488 nm laser), and **d** overlay image of the Hela cells on a field-effect transistor (FET) senor, which indicate that the •OH is generated intracellularly after addition of 10 μg mL^−1^ lipopolysaccharide (LPS). **e** Real-time response of the sensor (or pristine graphene) upon the •OH generated from the Hela cells after addition of 10 μg mL^−1^ LPS or 10 μg mL^−1^ LPS with 1% dimethyl sulfoxide. **f** Real-time response upon the •OH generated from the Hela cells after addition of 10 μg mL^−1^ LPS for the FET sensor modified by 10^−6^ M Zn^2+^ (black) or 10^−5^ M Zn^2+^ (red). The scale bars in **b**–**d** is 200 μm

The •OH plays an important role in the living system. As an application, the FET sensors are used for real-time detection of •OH produced by living cells within hours (Fig. 5a). Considering the bio-compatibility, Zn^2+^ was used as the indicator in the sensor. Hela cells were cultured on the surface of the graphene/Au/Cys-PP-Zn^2+^ FET sensor with interdigital source-drain electrodes (Fig. 5b, Supplementary Fig. 15). In the testing, lipopolysaccharide (LPS, 10 μg mL^−1^) was added as a stimulus to induce oxidative stress for the production of ROS within the Hela cells^9^. To verify the intercellular generation of ROS, the cells are incubated in 2′,7′-dichlorodihydrofluorescein diacetate (DCFH-DA) solution, which functions as the fluorescent probe for •OH. The probe is monitored by confocal microscopy upon 488 nm excitation. From the overlay image of the confocal fluorescence image and bright-field image (Fig. 5b–d), it is clear that the •OH has been generated in the Hela cells. As a comparison, no fluorescence (Supplementary Fig. 16) is observed without addition of LPS. Owing to the ultra-short lifetime, the intercellular •OH is hard to directly penetrate the cell membrane before converting to other kinds of ROS (i.e., H_2_O_2_, •O_2_^−^)^5^. Stable ROS (i.e., H_2_O_2_, •O_2_^−^) can penetrate the cell membrane, reach the solution environment^5^, and generate the extracellular •OH radicals. As a result, the graphene/Au/Cys-PP-Zn^2+^ FET device (modified by 10^−4^ M Zn^2+^) has a response in *I*_ds_ when the Hela cells are stimulated by LPS (Fig. 5e). The *I*_ds_ sharply increases in the first 1000 s and then maintains with a total change of current percentage of about 0.18%, in agreement with the response curve measured by other detection method^10^. When adding 1% dimethyl sulfoxide as the •OH scavenger, the total change of *I*_ds_ reduces to about 0.03 %, indicating the current signal is mainly from the •OH. Without addition of LPS or using the pristine graphene as the channel, no change of the *I*_ds_ is observed.

Quasi-quantitative detection of the •OH generated from the Hela cells is realized by using the FET sensors modified with different amount of Zn^2+^. The real-time current response to different concentrations of •OH (Supplementary Fig. 17) shows that the FET sensor (modified by 10^−5^ M Zn^2+^) has a small change of *I*_ds_ when exposing in 10^−7^ M •OH, while no response is observed in the case of the FET sensor modified by 10^−6^ M Zn^2+^. According to this result, the current response of different FET sensors upon the addition of 10 μg mL^−1^ LPS indicates the extracellular •OH concentration is at a level of ~10^−7^ M. This amount is lower than the intracellular concentration (10^−6^ M) measured by other methods^9–11^, in correspondence with the fact that the extracellular •OH originates from other ROS released from the cells and the production of •OH is relatively low (i.e., the rate constant of H_2_O_2_ + •O_2_^−^ → •OH + OH^−^ + O_2_ is around 1.3 M^−1^ s^−1^, <40–80 M^−1^ s^−1^ of Fenton reaction)^55,56^.

## Discussion

In this article, we realize the detection of •OH by an inner-cutting graphene FET sensor. Highly reactive •OH cuts the cysteamine to release Cd^2+^ from the electrolyte/graphene interface, leading to charge de-doping of the graphene channel. This, as well as the high carrier mobility and atomic thickness of graphene, result in high sensitivity of the FET sensor. By this strategy, •OH with a concentration down to 10^−9^ M is detectable, comparable or higher than some of the conventional free radical detection techniques (i.e., 10^−6^ M •OH for fluorescence spectroscopy)^9,11^. The inner-cutting strategy can not only monitor •OH but also has the potential to detect other types of highly reactive free radicals. Compared with other detection technologies like ESR spectroscopy and fluorescence spectroscopy, the inner-cutting graphene FET sensor is a portable and user-friendly analytical platform that realizes label-free real-time detection with both high selectivity and sensitivity. More importantly, based on the controllable charge doping and de-doping by metal ions, the •OH generated from the Hela cells can be quasi-quantitatively measured by using the graphene/Au/Cys-PP-Zn^2+^ FET sensors with different amount of Zn^2+^ dopant, indicating that the FET sensor is compatible with living system when non-heavy metal ions are used as the indicator, and the figure of merit still maintains when detecting in living system since 10^−7^ M extracellular •OH can be readily detected. This work extends the application of FET sensors in monitoring highly reactive free radical and realizes a real-time quasi-quantitative detection in living environment. Such a portable and user-friendly free radical FET sensor has great potential for practical applications in biological research, human health, environmental monitoring, etc.

## Methods

### Fabrication of the FET sensor

A monolayer graphene film was produced by CVD (Supplementary Note 1) and transferred onto a clean SiO_2_/Si substrate to fabricate a FET device by photolithography. The device was thermally annealed to improve the contacts between graphene and electrodes (50 nm Au/5 nm Cr), and then Au NPs were deposited onto graphene by vacuum evaporation (Covap, Angstrom Corp.) and thermal annealing at 200 °C for 30 min. The resulting graphene/Au NPs was submerged in 10^−2^ M cysteamine (Sigma-Aldrich) solution. The assembly of cysteamine on Au NPs was kept overnight at room temperature, followed by rinsing with ethanol for several times to remove extra cysteamine. The cysteamine-modified device was immersed in 10^−5^ M PP (Sigma-Aldrich) and *N*,*N*-dimethylformamide (DMF) solution for 15 h, by adding 1-ethyl-3-(3-dimethylaminopropyl) carbodiimide (Sigma-Aldrich) and *N*-hydroxysuccinimide (Sigma-Aldrich) as the catalysts. The resulting FET sensor, with immobilized PP, was rinsed with DMF, acetone, ethanol, and distilled water in sequence to remove extra residues. The graphene/Au/Cys-PP was dipped in Cd^2+^, Zn^2+^, or Mg^2+^ solution for 6 h before tests. Owing to the coordination reaction with PP, Cd^2+^, Zn^2+^, or Mg^2+^ was bound to the surface of FET to charge-dope graphene. A solution chamber, which was employed in all solution-based measurements, was fabricated by a three-dimensional printer.

### Characterization

The samples were measured by XPS (Perkin-Elmer PHI5300 with 250 W Mg Kα source), Raman (HORIBA XploRA, 532 nm layer), FESEM (Zeiss Ultra 55, acceleration voltage: 5 kV), TEM (Tecnai G2 F20 S-Twin, acceleration voltage: 200 kV), AFM (XE7, Parks System), and optical microscope (BH2, Olympus). The UV-vis absorption spectra (Lambda 750) were measured from the fluorine-doped tin oxide (FTO)-coated glass. The FTO-coated glass was thoroughly cleaned by sonication in the soapy water, ethanol, and 1.0 M KOH solution, followed by a rinse with doubly distilled water, and then it was dried with pure nitrogen stream and treated by the modification processes as graphene.

### Device measurement

The electrical signal was measured by a semiconductor parameter analyzer (Keysight, B1500A) and a probe station (Everbeing, PE-4) at room temperature in air. The •OH was generated through Fenton reaction by H_2_O_2_ and Fe^2+^ (H_2_O_2_/Fe^2+^ = 6:1). The electrolyte was 10^−4^ M PBS (0.01 × PBS, pH 7.2–7.4). All the aqueous solutions were prepared using distilled water with a resistivity of 18.2 MΩ cm. Ag/AgCl reference electrode was used as the liquid gate electrode. The *V*_gs_ was set lower than ±0.8 V (versus Ag/AgCl) and the *V*_ds_ was set to 50 mV to avoid any electrochemical reaction on the electrodes.

### Detection of the •OH generated by Hela cell

Hela cells were cultured on the FET sensors (Supplementary Note 4). The fluorescent images were captured by a laser confocal microscope (A42, Nikon) using 2 × 10^−6^ M DCFH-DA as the fluorescent probe. The device was immersed in 0.01 × PBS to keep the cells in good status and stored in a small culture dish for the measurement. Electrical measurement was performed by a semiconductor parameter analyzer (Keysight, B1500A) and a probe station (Everbeing, PE-4) without applying *V*_lg_ or *V*_bg_. After the *I*_ds_ became stable for 1000 s (*V*_ds_ = 50 mV), the electrolyte solution was quickly changed to 10 μg mL^−1^ LPS (0.01 × PBS) solution. The real-time *I*_ds_ was recorded for 4500 s in air.

## Supplementary information

Supplementary Information

## Data Availability

The data that support the findings of this study are available from the corresponding author upon request.
