# Supplementary material for: Free radical sensors based on inner-cutting graphene field-effect transistors
Source: Nat Commun. 2019 Apr 4;10:1544. doi: 10.1038/s41467-019-09573-4 (PMC6449349; doi:10.1038/s41467-019-09573-4)
Supplement: Supplementary file 1 — Supplementary Information [file 41467_2019_9573_MOESM1_ESM.pdf]

Supplementary Information for

"Free Radical Sensors Based on Inner-Cutting Graphene Field-Effect Transistor"

Zhen Wang,[1,2] Kongyang Yi,[1,2] Qiuyuan Lin,[3] Lei Yang,[1,2] Xiaosong Chen,[1,2] Hui Chen[3] & Dacheng Wei[1,2]*

[1] *State Key Laboratory of Molecular Engineering of Polymers, Fudan University, Shanghai 200433, China*

[2] *Department of Macromolecular Science, Fudan University, Shanghai 200433, China*

[3] *Department of Chemistry, Fudan University, Shanghai 200433, China*

* Corresponding author: weidc@fudan.edu.cn

Supplementary Note 1. Experimental details of preparation of graphene

Monolayer graphene was produced by chemical vapor deposition (CVD) as reported previously[1]. The graphene was grown on 25 μm thick Cu foils placed in the center of a tube furnace (GSL 1200X). The tube furnace was first heated to 1000 °C under a 4 sccm flow of H_2 (99.999%) within 30 min and kept at 1000 °C for 30 min. And then, 16 sccm CH_4 (99.999%) was introduced as the carbon source in the atmosphere of 4 sccm H_2. The growth process lasted for 15 min. Finally, the tube furnace was rapidly cooled to room temperature under the protection of H_2 gas, and the graphene film was obtained on the surface of Cu foil.

The graphene film was transferred to a clean SiO_2/Si wafer by the electrochemical bubbling method. The surface of the graphene-on-Cu was first coated with poly-methyl methacrylate (PMMA) by spin-coating. And then, the graphene film was removed from the Cu foils by electrochemical bubbling at a potential of 2.5 V in 0.5 M NaOH aqueous solution. The PMMA/graphene was released from Cu substrate and washed by distilled water for three times, and then was lifted from the solution and transferred on the surface of clean SiO_2/Si. The graphene film on SiO_2/Si was obtained by further annealing in Ar (99.999%) at 400 °C for 30 min to remove the PMMA.

Supplementary Note 2. Calculation of the surface charge density

According to the literature[2], the surface charge density on the graphene surface can be calculated by the equation

$$\Delta n = C_{\mathrm{lq}}\Delta V_{\mathrm{Dirac}}/q \qquad (1)$$

where Δn is the change of surface charge density. C_{lq} is the total interfacial capacitance between electrolyte and graphene, which consists of the double layer capacitance and the quantum capacitance[3]. The C_{lq} is around 3 μF cm^{-2}, as previously reported[4]. $\Delta V_{\mathrm{Dirac}}$

is the Dirac point shift and q is the elementary charge (1.9×10^{-19} C). The ΔV_{Dirac} is extracted from Fig. 2b to be about −420 mV after the device is treated with 10^{-4} M Cd^{2+}, and to be about 57 mV after exposing the device in 10^{-4} M •OH. According to the equation, Δn is calculated to be about 0.1 nm^{-2}. Owing to the high-quality of the CVD graphene, we assume the charge density of the pristine graphene surface is 0 nm^{-2}. After modifying the porphyrin on the graphene surface in 10^{-4} M Cd^{2+}, the surface charge density increases to about 0.1 nm^{-2} (about 10 Cd^{2+} on every 100 nm^2 graphene surface). After exposure in 10^{-4} M •OH, the surface charge density decreases by 0.0136 nm^{-2} (about 1.36 Cd^{2+} on every 100 nm^2 graphene surface are removed).

Supplementary Note 3. Debye length

The Debye Length (λ_d) is one of the key parameters in liquid gated FET sensor. Within λ_d, the charged target molecules will introduce a current response in the conducting channel. If the distance between the charged target molecules and the channel is larger than λ_d, a significant deterioration of the sensor performance will be expected[5]. Here, the λ_d in 0.01×PBS is around 7.3 nm. In the FET sensor, the distance between the indicators (Cd^{2+}, Zn^{2+} or Mg^{2+}) and the graphene surface is ~6 nm, according to the AFM result in Supplementary Fig. 2. The value is smaller than the λ_d, allowing the sensitive response of the FET sensor upon the change of the amount of metal ion indicators on the graphene surface.

Supplementary Note 4. Cell culture and fluorescent imaging.

Hela cells were cultured in Dulbecco’s modified Eagle’s medium (DMEM) including high glucose with 10% (v/v) fetal bovine serum, penicillin (100 units mL^{-1}), and streptomycin (100 μg mL^{-1}). The cells were seeded in a 25 cm^2 culture bottle and incubated in the incubator for 12 h in an atmosphere of 5% CO_2 and 95% air at 37ºC.

To culture the Hela cell on the FET sensor, the device was put into a culture bottle, and then the Hela cells were seeded and incubated on the device in the same condition.

To capture a fluorescent image, a device on glass substrate with Hela cells was incubated in 2 μM DCFH-DA solution for 20 min. After washing the extra DCFH-DA by PBS, the device was incubated in 10 μg mL^{-1} LPS for 30 min. The Hela cells generated the •OH, which reacted with the DCFH-DA fluorescent probe. By using a confocal microscopy, the probe was monitored and a fluorescent image (525 nm) was captured upon 488 nm laser excitation.

Supplementary Fig. 1

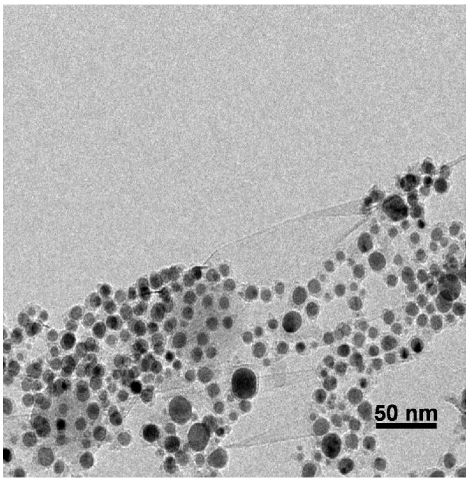

Supplementary Fig. 1. TEM image of Au NPs on graphene.

Supplementary Fig. 2

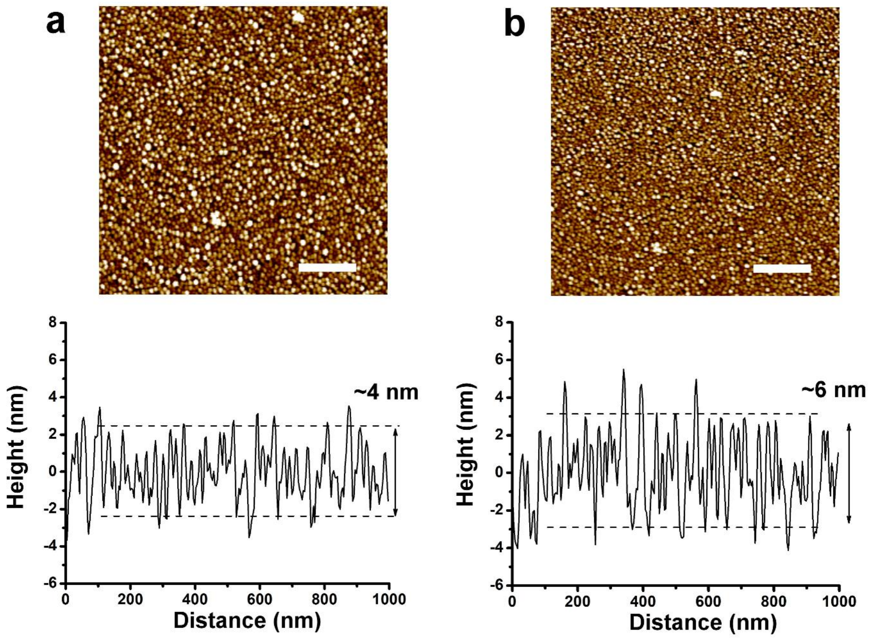

Supplementary Fig. 2. AFM images of the graphene/Au NPs. The images are obtained **a,** before and **b,** after modification with cysteamine and protoporphyrin IX. The lower insets are the height profile across the surface of the graphene/Au NPs from the AFM images. The scale bars are 200 nm.

Supplementary Fig. 3

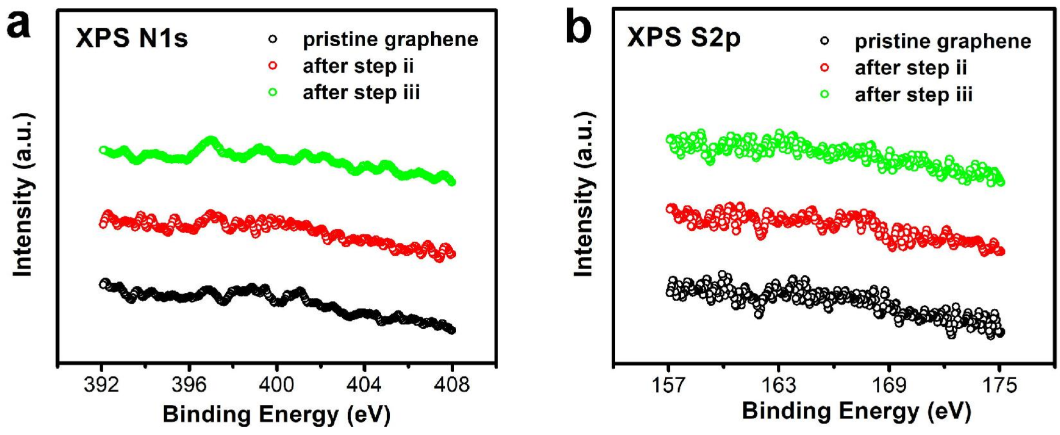

Supplementary Fig. 3. XPS of the sample. **a,** N1s and **b,** S2p spectra of the pristine graphene (black), the graphene after step ii (red, graphene/Cys) and after step iii (green, graphene/Cys-PP) without depositing the Au NPs.

Supplementary Fig. 4

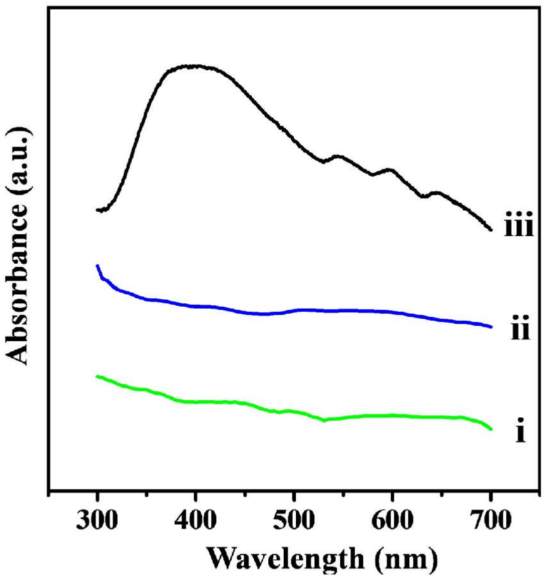

Supplementary Fig. 4. UV-vis absorption spectra of (ⅰ) FTO/Au, (ⅱ) FTO/Au/Cys, and (ⅲ) FTO/Au/Cys-PP.

Supplementary Fig. 5

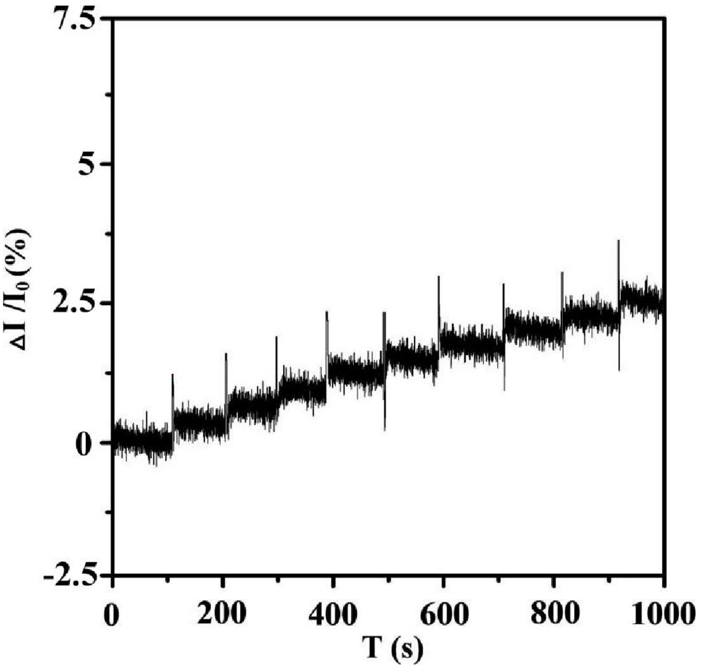

(a)

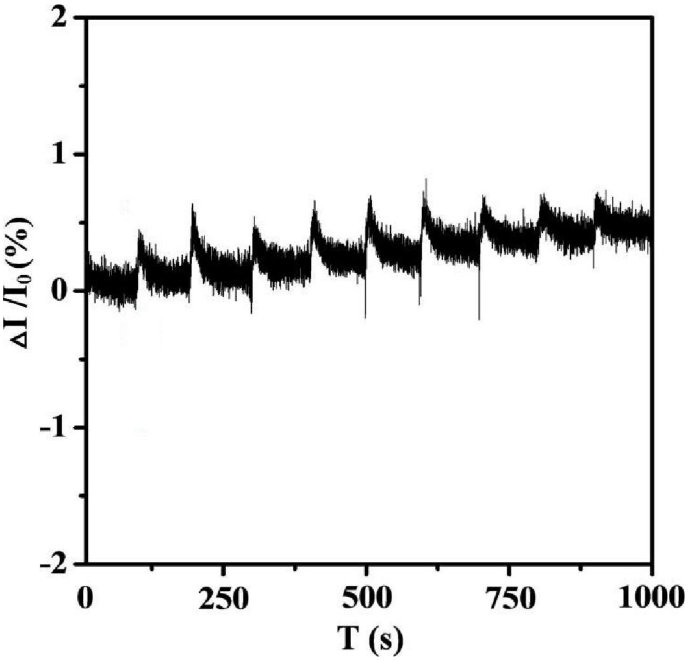

(b)

Supplementary Fig. 5. Real-time electrical measurement of a FET sensor (modified by Cd^{2+}) upon addition of **a,** 1×10^{-6} M •OH or **b,** 1×10^{-7} M •OH.

Supplementary Fig. 6

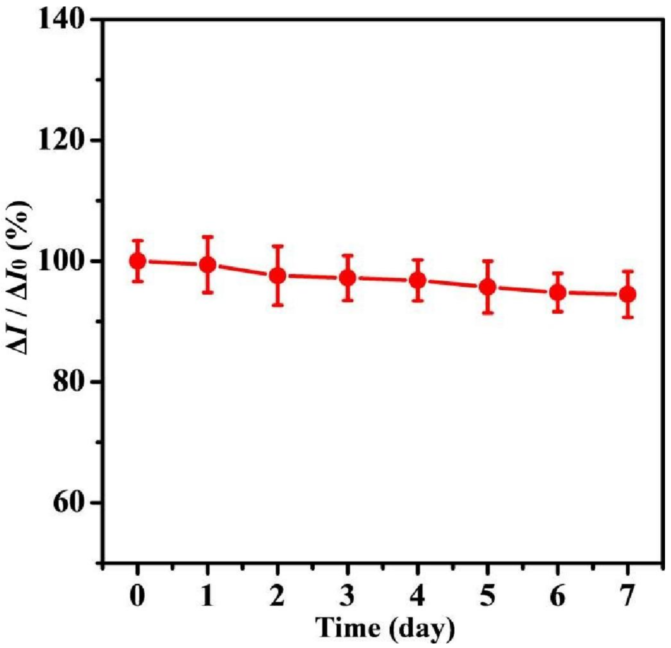

Supplementary Fig. 6. Stability test of the graphene/Au/Cys-PP-Cd^{2+} sensor for 7 days. The current response (ΔI) of the FET device to 10^{-4} M •OH was monitored three times daily after stored in a refrigerator, and the ΔI when adding 10^{-4} M •OH still retains more than 94.5% of its initial response (ΔI_0) after one week storage. The error bars are defined by the standard deviation of the results from three parallel experiments.

Supplementary Fig. 7

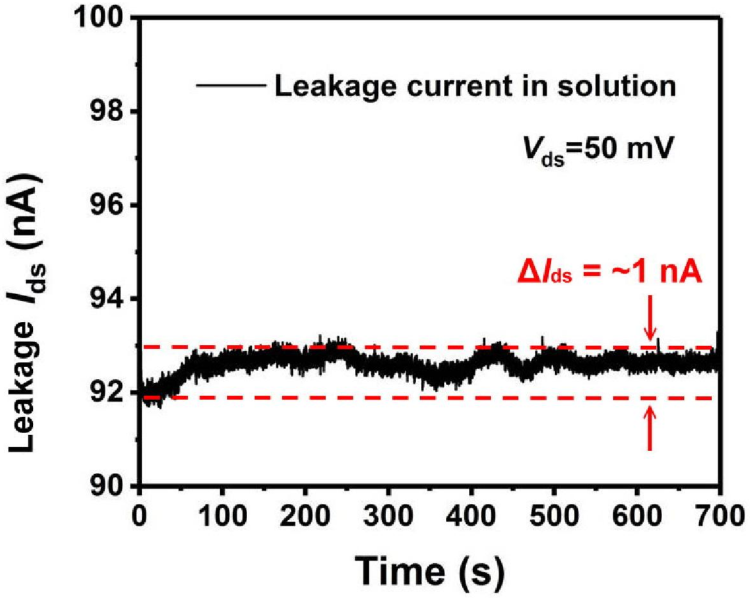

Supplementary Fig. 7. The leakage current of the device in the solution. The curve shows the leakage I_{ds} current versus time when the bare interdigital electrodes are immersed in the 0.01×PBS solution. The leakage I_{ds} is tiny compared with I_{ds} (200 μA or higher at V_{ds} = 50 mV). The leakage I_{ds} fluctuation is only around 1 nA, smaller than 0.0005% of the I_{ds}, thus it can be ignored in the measurement.

Supplementary Fig. 8

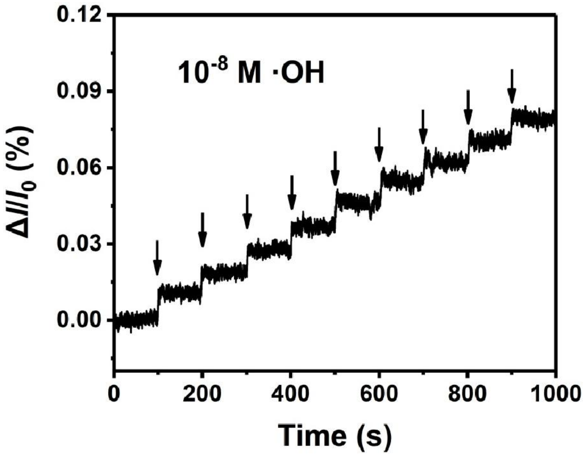

(a)

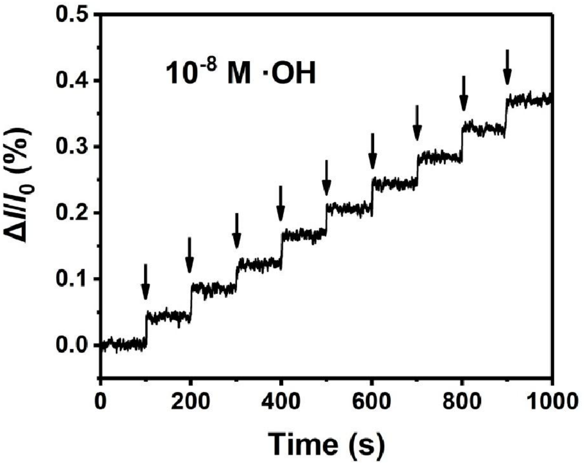

(b)

Supplementary Fig. 8. Real-time electrical measurement of **a,** the graphene/Au/Cys-PP-Cd^{2+} FET sensor and **b,** the graphene/Au/Cys-PP-Mg^{2+} FET sensor upon addition of 10^{-8} M •OH. The sensor used interdigital electrodes (50 μm channel length), and was modified in **a,** 10^{-4} M Cd^{2+} or **b,** 10^{-4} M Mg^{2+}.

Supplementary Fig. 9

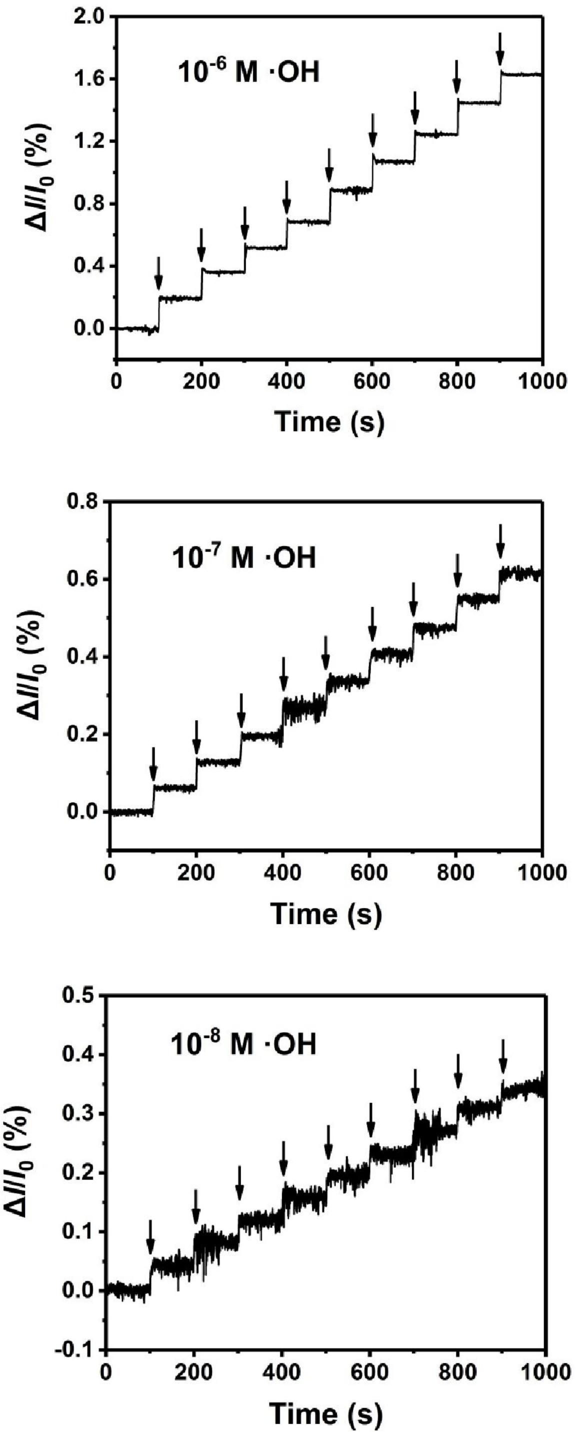

(a) (b) (c)

Supplementary Fig. 9. Real-time electrical measurement of the graphene/Au/Cys-PP-Zn^{2+} FET sensor upon addition of **a,** 10^{-6} M, **b,** 10^{-7} M or **c,** 10^{-8} M •OH. The sensor used interdigital electrodes (50 μm channel length), and was modified in 10^{-4} M Zn^{2+}.

Supplementary Fig. 10

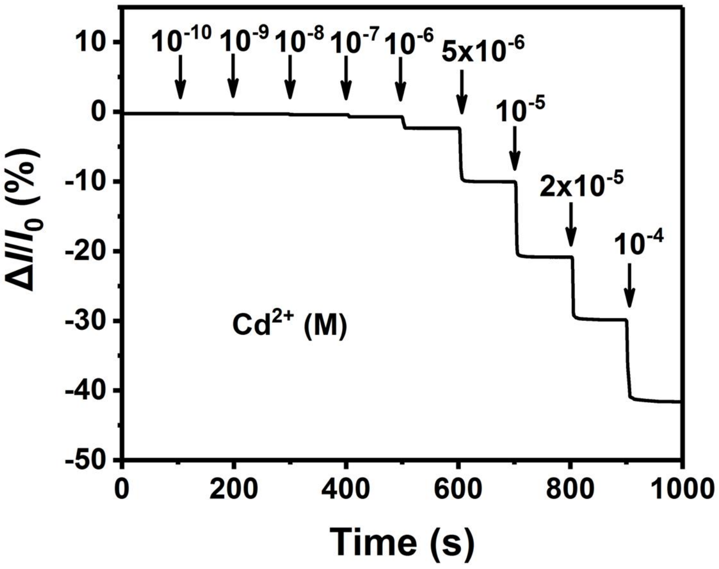

(a)

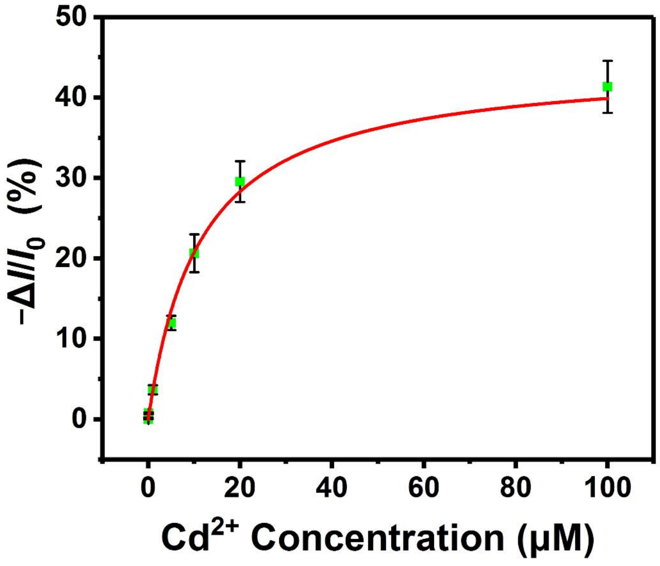

(b)

Supplementary Fig. 10. Current response of the graphene/Au/Cys-PP to the Cd^{2+}. **a,** Real-time electrical measurement of the graphene/Au/Cys-PP FET sensor upon addition of 10^{-10} ~ 10^{-4} M •OH. **b,** Current response versus the Cd^{2+} concentration. The sensor used interdigital electrodes (50 μm channel length). The error bars are defined by the standard deviation of the results from three parallel experiments.

Supplementary Fig. 11

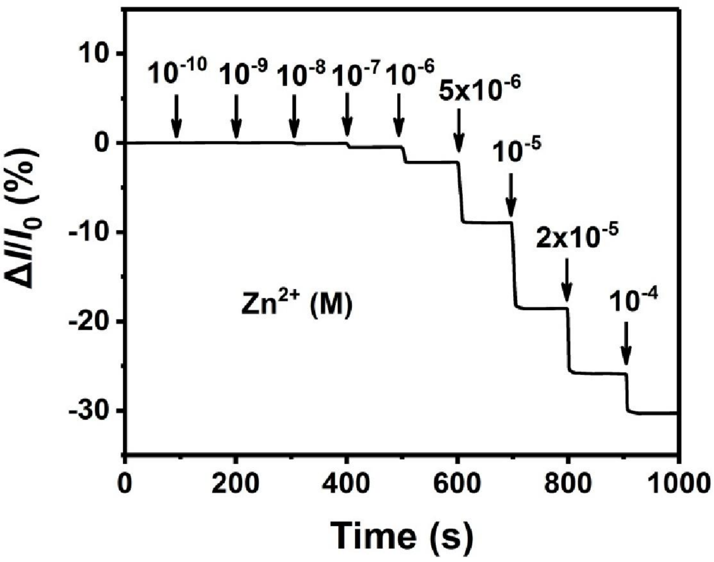

(a)

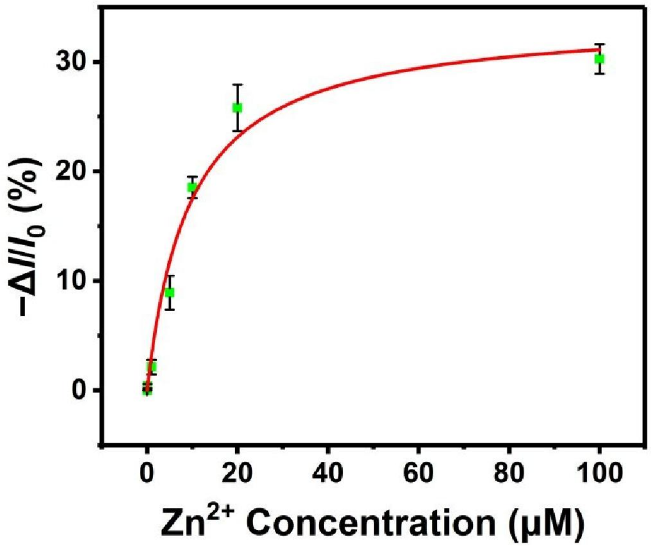

(b)

Supplementary Fig. 11. Current response of the graphene/Au/Cys-PP to the Zn^{2+}. **a,** Real-time electrical measurement of the graphene/Au/Cys-PP FET sensor upon addition of 10^{-10} ~ 10^{-4} M •OH. **b,** Current response versus the Zn^{2+} concentration. The sensor used interdigital electrodes (50 μm channel length). The error bars are defined by the standard deviation of the results from three parallel experiments.

Supplementary Fig. 12

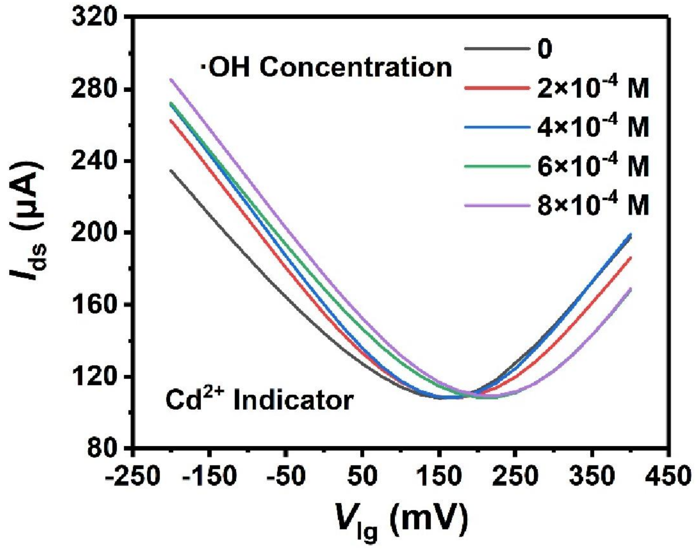

(a)

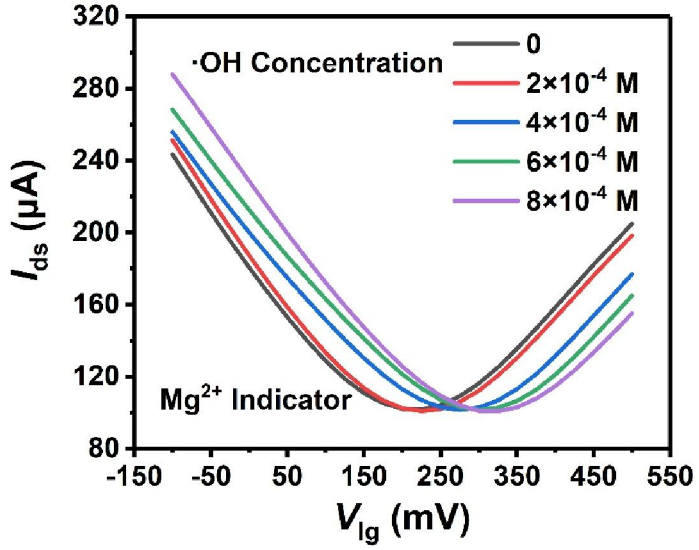

(b)

Supplementary Fig. 12. Liquid gate transfer curve of the FET sensor. Liquid gate transfer curves (V_{ds} = 50 mV) of **a,** a graphene/Au/Cys-PP-Cd^{2+} FET device (modified in 10^{-4} M Cd^{2+}) and **b,** a graphene/Au/Cys-PP-Mg^{2+} FET device (modified in 10^{-4} M Mg^{2+}) before and after addition of 2×10^{-4}, 4×10^{-4} M, 6×10^{-4} M and 8×10^{-4} M •OH. The sensors used interdigital electrodes (50 μm channel length).

Supplementary Fig. 13

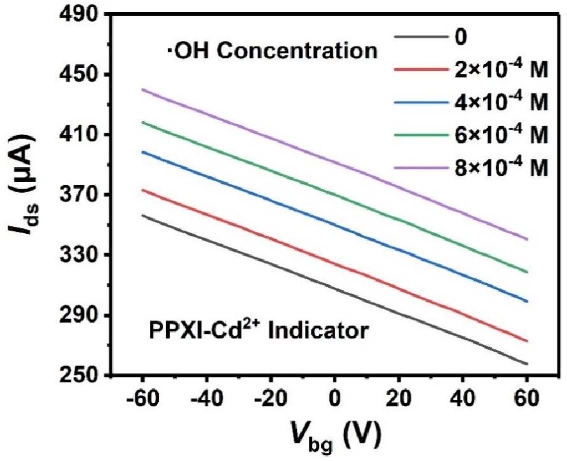

(a)

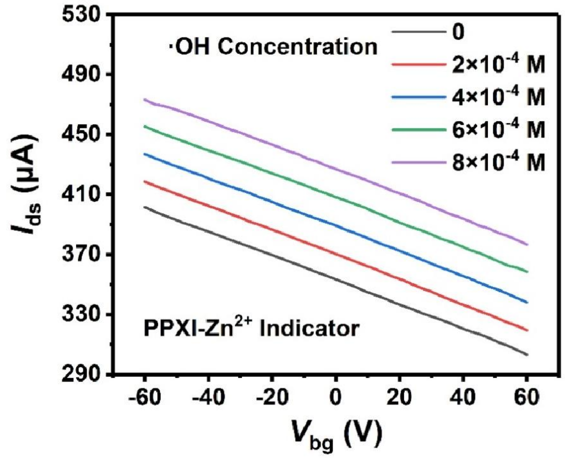

(b)

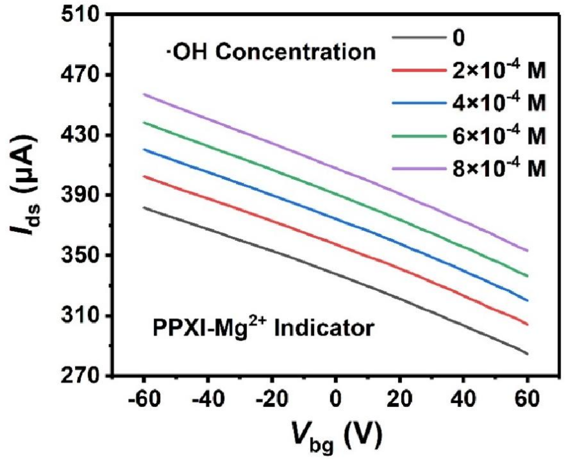

(c)

Supplementary Fig. 13. Back gate transfer curve of the FET sensor. Back gate transfer curves (V_{ds} = 50 mV) of **a,** a graphene/Au/Cys-PP-Cd^{2+} FET device (modified in 10^{-4} M

Cd^{2+}) and **b,** a graphene/Au/Cys-PP-Zn^{2+} FET device (modified in 10^{-4} M Zn^{2+}) and **c,** graphene/Au/Cys-PP-Mg^{2+} FET device (modified in 10^{-4} M Mg^{2+}) before and after addition of 2×10^{-4}, 4×10^{-4} M, 6×10^{-4} M and 8×10^{-4} M •OH. The sensors used interdigital electrodes (50 μm channel length).

Supplementary Fig. 14

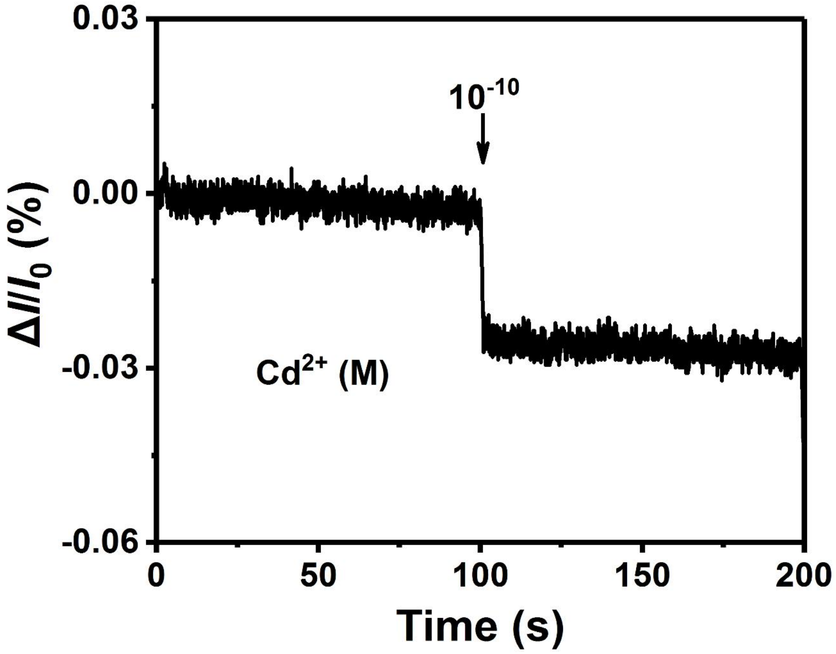

Supplementary Fig. 14. Real-time electrical measurement of graphene/Au/Cys-PP FET sensor upon addition of 10^{-10} M Cd^{2+}. The sensor used interdigital electrodes (50 μm channel length).

Supplementary Fig. 15

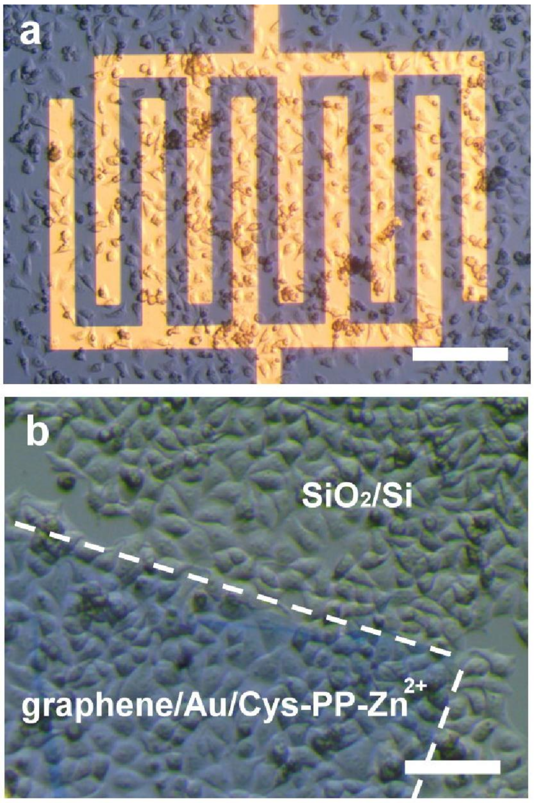

Supplementary Fig. 15. Optical microscope images of the FET devices with Hela cells. Hela cells were cultured on the surface of the graphene/Au /Cys-PP-Zn^{2+} FET with interdigital source-drain electrodes. The scale bars are 200 μm in **a**, and 100 μm in **b**.

Supplementary Fig. 16

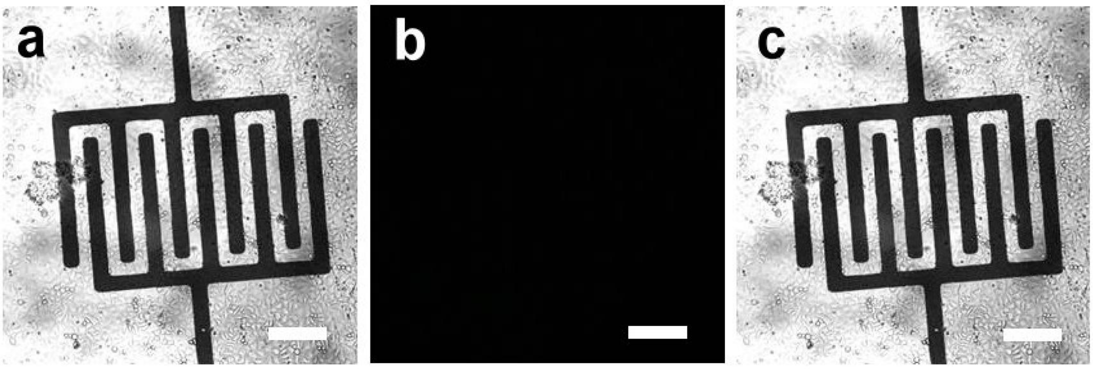

Supplementary Fig. 16. Optical microscope images of a FET device. **a,** Bright field image, **b,** confocal fluorescence image (excited by 488 nm laser) and **c,** overlay image of the Hela cells on a FET senor. DCFH-DA is used as the fluorescent probe for •OH. Without addition of LPS, no fluorescence was observed. The scale bars are 200 μm.

Supplementary Fig. 17

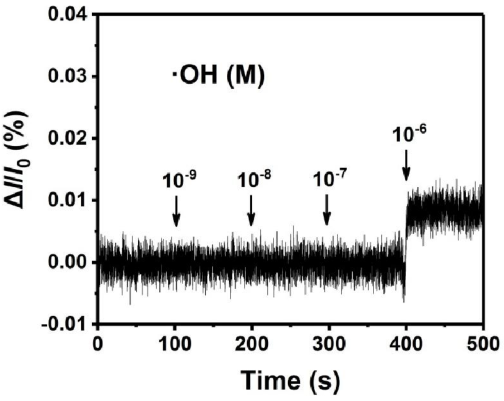

(a)

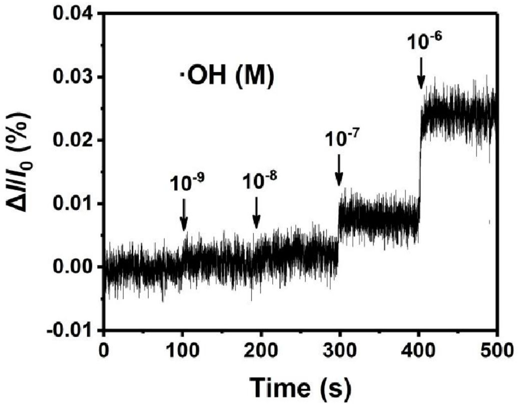

(b)

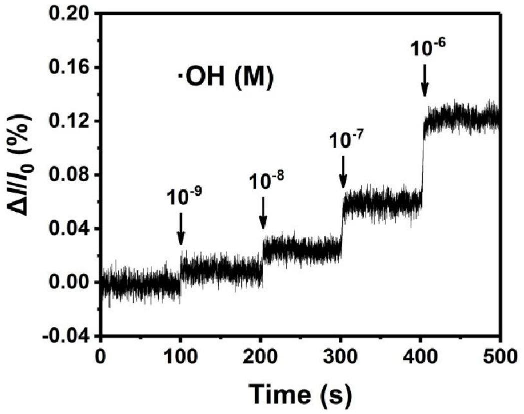

(c)

Supplementary Fig. 17. Real-time response upon various concentrations of •OH (from 10^{-9} to 10^{-6} M) for the FET sensors with channel modified with **a,** 10^{-6} M Zn^{2+}, **b,** 10^{-5} M Zn^{2+} or **c,** 10^{-4} M Zn^{2+}. The sensors used interdigital electrodes (50 μm channel length).

Supplementary References

1. Li, X. S., Cai, W. W., An, J. H., Kim, S., Nah, J., Yang, D. X., Piner, R., Velamakanni, A., Jung, I., Tutuc, E., Banerjee, S. K., Colombo, L., Ruoff, R. S. Large-area synthesis of high-quality and uniform graphene films on copper foils. *Science* **324,** 1312–1314 (2009).

2. Yamamoto, M., Ueno, K. & Tsukagoshi, K. Pronounced photogating effect in atomically thin WSe_2 with a self-limiting surface oxide layer. *Appl. Phys. Lett.* **112,** 181902 (2018).

3. Ohno, Y., Maehashi, K., Yamashiro, Y. & Matsumoto, K. Electrolyte-gated graphene field-effect transistors for detecting pH and protein adsorption. *Nano Lett.* **9,** 3318–3322 (2009).

4. Hess, L. H. et al. High-transconductance graphene solution-gated field effect transistors. *Appl. Phys. Lett.* **99,** 033503 (2011).

5. Stern, E. *et al.* Importance of the Debye Screening Length on Nanowire Field Effect Transistor Sensors. *Nano Lett.* **7**, 3405–3409 (2007).
